# NUC041, a Prodrug of the DNA Methytransferase Inhibitor 5-aza-2′,2′-Difluorodeoxycytidine (NUC013), Leads to Tumor Regression in a Model of Non-Small Cell Lung Cancer

**DOI:** 10.3390/ph11020036

**Published:** 2018-04-23

**Authors:** Richard Daifuku, Sheila Grimes, Murray Stackhouse

**Affiliations:** 1 Epigenetics Pharma, 9270 SE 36th Pl, Mercer Island, WA 98040, USA; 2 Southern Research, 2000 9th Avenue South, Birmingham, AL 35205, USA; sgrimes@southernresearch.org (S.G.); mstackhouse@southernresearch.org (M.S.)

**Keywords:** 5-azacytidine, cancer, decitabine, epigenetics, DNA methyltransferase, natural killer cells, NUC013, NUC041, nucleoside, p53, ribonucleotide reductase

## Abstract

5-aza-2′,2′-difluorodeoxycytidine (NUC013) has been shown to be significantly safer and more effective than decitabine in xenograft models of human leukemia and colon cancer. However, it suffers from a similar short half-life as other DNA methyltransferase inhibitors with a 5-azacytosine base, which is problematic for nucleosides that primarily target tumor cells in S phase. Because of the relative instability of 5-azanucleosides, a prodrug approach was developed to improve the pharmacology of NUC013. NUC013 was conjugated with trimethylsilanol (TMS) at the 3′ and 5′ position of the sugar, rendering the molecule hydrophobic and producing 3′,5′-di-trimethylsilyl-2′,2′-difluoro-5-azadeoxycytidine (NUC041). NUC041 was designed to be formulated in a hydrophobic vehicle, protecting it from deamination and hydrolysis. In contact with blood, the TMS moieties are readily hydrolyzed to release NUC013. The half-life of NUC013 administered intravenously in mice is 20.1 min, while that of NUC013 derived from intramuscular NUC041 formulated in a pegylated-phospholipid depot is 3.4 h. In a NCI-H460 xenograft of non-small cell lung cancer, NUC013 was shown to significantly inhibit tumor growth and improve survival. Treatment with NUC041 also led to significant tumor growth inhibition. However, NUC041-treated mice had significantly more tumors ulcerate than either NUC013 treated mice or saline control mice, and such ulceration occurred at significantly lower tumor volumes. In these nude mice, tumor regression was likely mediated by the derepression of the tumor suppressor gene p53 and resultant activation of natural killer (NK) cells.

## 1. Introduction

The central feature of cancer is increasingly considered to be an unstable and disrupted epigenome, i.e., the epigenetic information in a cell, comprising DNA methylation, post-translational modifications of histones, and higher-order chromatin structure [1]. DNA methyltransferase (DNMT) inhibitors are a class of drugs used in the treatment of cancer that aim to express aberrantly silenced genes through hypermethylation, e.g., genes associated with reduced proliferation, cell differentiation, apoptosis, and senescence. Decitabine and 5-azacytidine (5-azaC) remain the only approved drugs of this class but suffer from relative toxicity and poor stability [2]. There are non-aza nucleosides under clinical development as DNMT inhibitors, but these are characterized by relatively weak DNMT inhibition and even shorter half-lives [3,4].

Decitabine’s half-life in humans is reported to be 35 min by IV infusion [5]. Because decitabine is a cell cycle specific agent, a 1 to 4 h infusion of this agent only targets cancer cells in S phase, whereas cells in G1 and G2 escape the chemotherapeutic action of this analog during short-term treatment [6]. Attempts have been made to improve efficacy through continuous infusion, allowing greater incorporation into DNA; however, these have been hampered by inconvenience and toxicity [7,8]. 

**Figure 1:**
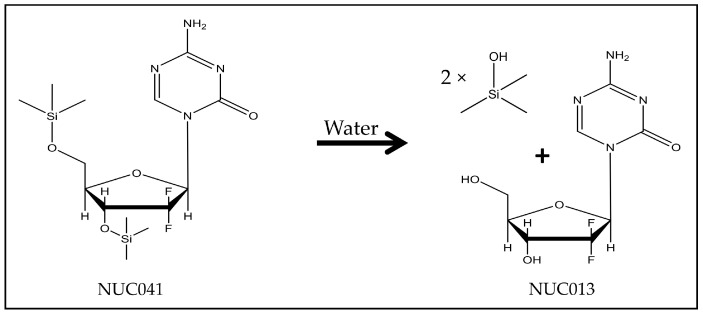
Hydrolysis of NUC041 to NUC013 with release of TMS.

2′-2′-difluoro-5-azadeoxycytidine (NUC013) is a DNA methyltransferase (DNMT) inhibitor and a ribonucleotide reductase (RNR) inhibitor. It has been shown to significantly inhibit growth in more tumor cell lines than decitabine in the NCI 60 cell line panel. Furthermore, NUC013 has been shown to be safer and more effective than decitabine in xenograft models of human leukemia (HL-60) and colon cancer (LoVo) [9]. However, NUC013 suffers from the same instability and short half-life as other nucleosides with a 5-azacytosine base, likely as a result of deamination by cytidine deaminase (CDA) [10]. Because known cytidine prodrug motifs, such as conjugation of a carbamate at the N-4 amino [11,12] would further destabilize the 5-azacytosine base, which is hydrolytically cleaved between the C-5 and C-6, a new prodrug approach was taken, one that is formulation dependent. A hydrophobic prodrug was developed for packaging in a hydrophobic vehicle to protect NUC013 from hydrolysis and deamination. In an aqueous environment, the carrier moieties were readily hydrolyzed with release of NUC013. This was achieved by conjugating NUC013 with trimethylsilanol (TMS) at the 3′ and 5′ position of the sugar moiety to form 3′,5′-di-trimethylsilyl-2′,2′-difluoro-5-azadeoxycytidine (NUC041) (Figure 1). TMS has previously been proposed as a prodrug moiety for various cytidine analogs [13], and there are examples of silyl prodrugs proposed in the literature; however, none have reported validation through in vivo testing [14,15].

Presented below are results of a series of experiments with NUC041, leading to a formulation providing a prolonged half-life with concomitant improvement in the outcome of tumor treatment in a model of non-small cell lung cancer (NSCLC). 

## 2. Results

### 2.1. In Vitro Comparison of 5-azaC or NUC013 with Its Respective TMS Prodrug

**Figure 2:**
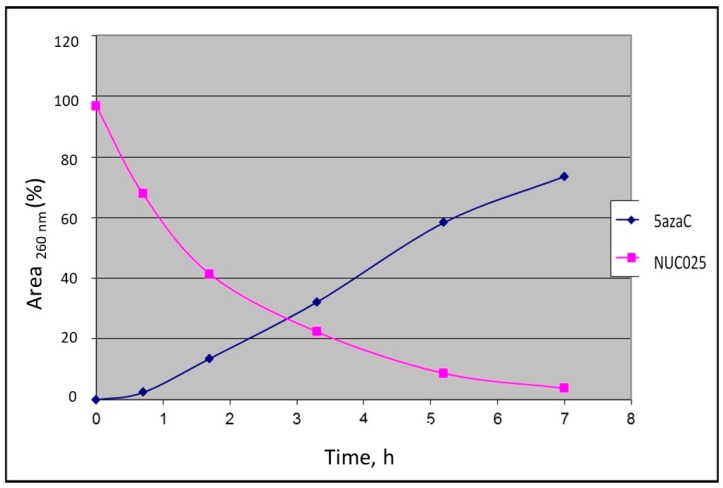
Hydrolysis of NUC025 in saline with release of 5-azaC at 20 °C measured by spectrophotometry.

Initial feasibility studies were performed with the TMS prodrug of 5-azaC for ease of synthesis (NUC025, 2′,3′,5′-tri-trimethylsilyl-5-azacytidine). Using a spectrophotometer, absorbance was measured at 260 nm and percent area of time 0 calculated and thereafter fractions thereof. At 20 °C in saline, NUC025 efficiently released 5-azaC by hydrolysis (Figure 2).

To confirm these results, the GI_50_ of NUC013 was compared to that of NUC041 in two cancer cell lines: human non-small cell lung cancer (NSCLC) NCI-H460 and colon cancer HCT-116 (Table 1).

The GI_50_ of NUC041 was comparable to that of NUC013, confirming efficient release of the nucleoside from the prodrug in vitro. 

### 2.2. In Vivo Studies with NUC041 Formulated in a Lipid Nano-Emulsion

The pharmacokinetics of NUC041 and NUC013 were determined in mouse whole blood, with NUC041 formulated in a lipid nano-emulsion (LNE) administered IV at a dose of 15 mg/kg (Table 2).

The half-life of NUC013 released from NUC041 formulated in LNE was comparable to that from NUC013 reconstituted in saline and administered IV, 20.1 min [9]. 

**Figure 3:**
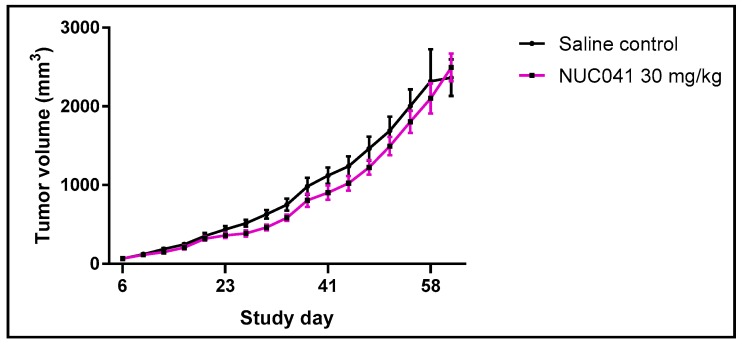
Comparison of mean tumor volumes (±SEM) in mice with human colon cancer (LoVo) implants treated with NUC041 30 mg/kg IV administered three consecutive days per week for three weeks vs. SC. (*n* = 10 per group).

NUC041 in LNE was tested at 30 mg/kg IV for three consecutive days a week for three weeks in a mouse xenograft model of human colon cancer (Lovo) and compared to saline control (SC). Growth of LoVo in mice treated with NUC041 was significantly inhibited compared to SC on study days 27–30 (*p* < 0.05, Student *t*-test, two tailed) (Figure 3).

### 2.3. Pharmacokinetic Studies of NUC041 Formulated in PEG-Phospholipid Depot

**Figure 4:**
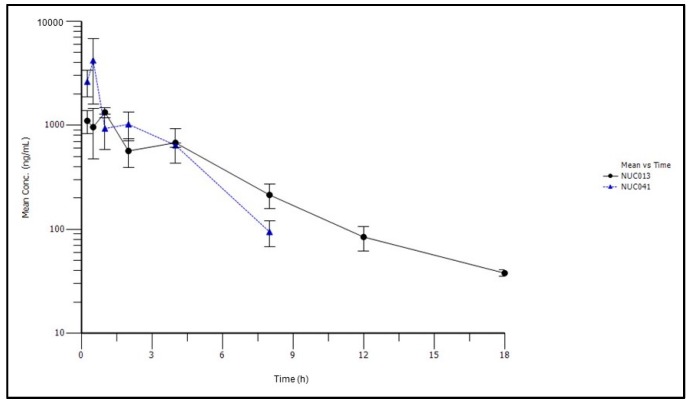
Mean concentrations (±standard deviation) of NUC041 and NUC013 in blood over time following IM administration of NUC041 (3 mg/mouse) in PPD vehicle.

Subsequently, NUC041 was formulated in a PEG-phospholipid depot (PPD) for intramuscular (IM) injection in an attempt to improve the half-life. In all studies with NUC041 formulated in PPD, a fixed dose was administered to each mouse because the volume of drug was too small to allow adjustments based on animal weight and the syringes used could only administer drug in increments of 10 µL. Thus, NUC041 was administered at a fixed dose of 3 mg (50 µL) to mice with a mean weight of 31.9 g (mean dose of 90.4 mg/kg). Results of the pharmacokinetic analysis are presented in Table 3 and Figure 4.

The half-life of NUC013 increased from 15 min when NUC041 was formulated in LNE to 3.4 h when PPD was used as vehicle. The Vss decreased from 4431 mL/kg to a Vz/F of 1172 mL/kg, and the AUC_inf_ on a mg/kg basis increased by 4.1-fold.

### 2.4. Tolerability Studies of NUC041 Formulated in PPD

Administration of 50 µL of PPD vehicle IM led to a weight loss of 10.9% two days following vehicle administration. NUC041 was formulated at a concentration of 60 mg/mL in this vehicle. The maximum tolerated dose (MTD) was 0.6 mg IM every other day (qod) per mouse corresponding to 10 µL of vehicle (mean mouse weight at study initiation of 23.4 g, corresponding to a mean dose is 25.6 mg/kg). Administration of 50 µg of intraperitoneal (IP) dexamethasone 30 min prior to formulated NUC041 allowed IM injection of a dose of 3 mg per mouse (mean mouse weight 24.2 g at study initiation, corresponding to a mean dose of 123.8 mg/kg) without weight loss or lethality. 

### 2.5. Human NSCLC NCI-H460 Xenograft Treated with NUC013 and NUC041 in PPD

**Figure 5:**
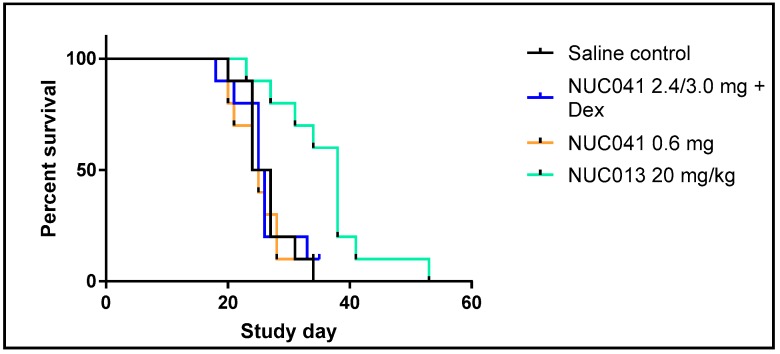
Survival proportions of NUC013, NUC041 with dexamethasone (Dex) pretreatment, and NUC041 vs. SC (*n* = 10 per group) in mice with human NSCLC NCI-H460 tumor implants. Survival refers to animals that were not removed from the study by death or per protocol euthanasia. Mice were administered the following doses and regimens: NUC013 20 mg/kg IV qd for three consecutive days per week (qwk) × 4; NUC041 2.4 mg/mouse IM qwk × 3 then 3.0 mg/mouse IM qwk × 1, with 50 µg dexamethasone IP 30 min prior to NUC041 injection; NUC041 0.6 mg IM qod × 15. Median survival (MS) SC 25.5 days. (1) NUC013, MS = 38 days, hazard ratio (HR) = 0.14 (*p* = 0.0018, Log rank test); (2) NUC041 + Dex, MS = 25.5 days (*p* = NS); NUC041 0.6 mg, MS = 24.5 days (*p* = NS).

As per Figure 5, mice treated with NUC013 20 mg/kg IV for three consecutive days a week for four weeks had significantly improved survival vs. SC. Median survival of SC mice was 25.5 days vs. 38 days for mice treated with NUC013 (hazard ratio (HR) = 0.14, *p* = 0.0018). However, no survival benefit was noted for mice treated with NUC041 with or without dexamethasone pretreatment. This lack of survival benefit was related to per protocol euthanasia of mice with ulcerated tumors. The last surviving mouse in the NUC041 with dexamethasone group was euthanized on day 35 for histology as was the last surviving mouse in the NUC041 0.6 mg group on day 34.

**Figure 6:**
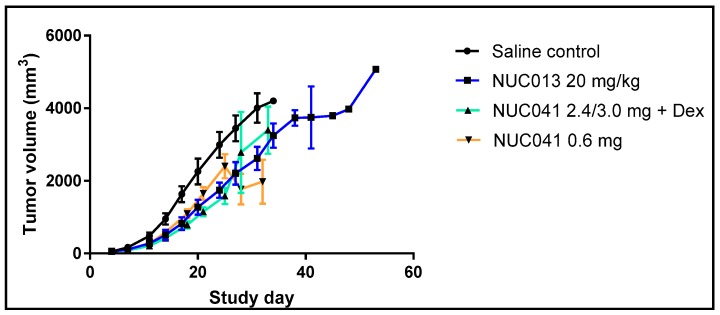
Comparison of mean tumor volumes (±SEM) in mice with human NSCLC NCI-H460 implants. Drug dose, route, and regimen as per Figure 5. Two mice in the NUC041 0.6 mg group had ulcerated tumors on study day 28 and should have been euthanized per protocol, but, by amendment, received additional doses of study drug on days 30 and 32 and were euthanized on study day 34 (*n* = 10 per group).

As per Figure 6, mice treated with NUC013 had significantly lower tumor volumes vs. SC on study days 14 through 27 (*p* < 0.05, Student *t*-test, 2-tailed). Mice with NUC041 with dexamethasone pretreatment had significantly lower tumor volumes vs. SC on study days 7 through 25, while mice in the NUC041 0.6 mg group had significantly lower tumor volumes vs. SC on study days 11 through 18 and again on day 28 (both, *p* < 0.05, Student *t*-test, 2-tailed). The dip in tumor volumes on study day 28 in the NUC041 0.6 mg group was partially related to tumor regression noted in two mice. One mouse had regression in tumor volume from day 25 to 32 of 38% and the other of 51%.

### 2.6. Tumor Histology

Table 4 summarizes the findings in mice that had tumor histology. 

**Figure 7:**
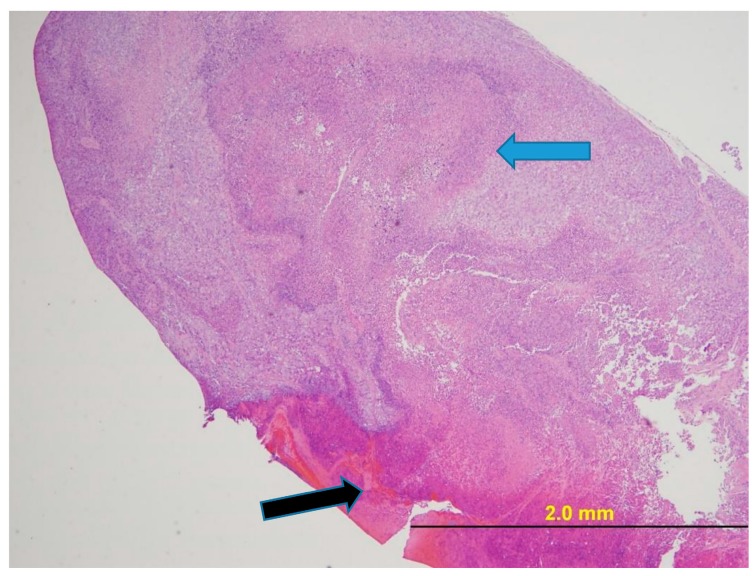
NCI-H460 human NSCLC xenograft (Mouse 3 with ulcerated 1960 mm^3^ tumor). H&E stain. The tumor had foci of necrosis consisting of necrotic neoplastic epithelial cells and degenerate and karyorrhectic neutrophils (blue arrow). The surface of the tumor was ulcerated (black arrow). At the margin of the neoplasm, within a band of fibrous connective tissue, neutrophils were admixed with several lymphocytes and plasma cells.

**Figure 8:**
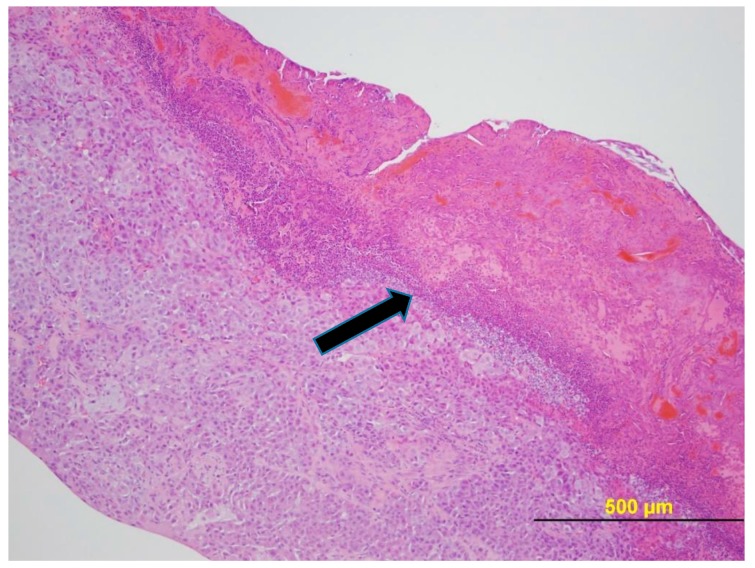
NCI-H460 human NSCLC xenograft (Mouse 4 with ulcerated 936 mm^3^ tumor). H&E stain. The tumor had an ulcerated surface with karyorrhectic neutrophilic debris, eosinophilic debris, hemorrhage, keratin, and necrotic neoplastic cells within the site of ulceration (black arrow).

Figure 7 provides a photomicrograph of the tumor from Mouse 3 and Figure 8 from Mouse 4 as illustrative of histology of mice with ulcerated tumors. In both cases, once ulceration is excluded as the tumor examined had only a small area of neoplasia. The tumors peaked in volume at 3179 mm^3^ for the 1960 mm^3^ tumor (38% regression) and at 1913 mm^3^ for the 936 mm^3^ tumor (51% regression).

## 3. Discussion

The prodrug technology of conjugating TMS with NUC013 at the 3′ and 5′ positions was specifically developed to protect a 5-aza nucleoside from deamination and hydrolysis. Extensive toxicology has been performed on TMS. At high doses, TMS is a central nervous system depressant, producing sedation, hypnosis, and general anesthesia in the rat, guinea pig, and rabbit. Depression was the only effect observed in studies in which TMS was administered by oral, subcutaneous (SQ), IM, IP, or IV routes, and this effect was reversible. Doses in the range of 100–200 mg/kg IV produced light to moderate anesthesia, persisting for 10 to 60 min. Oral subchronic and chronic studies at doses of up to 250 mg/kg showed no significant toxic effects. TMS administered orally to the rat was absorbed and eliminated within 48 h from the body [16].

An in vitro study with NUC025 demonstrated hydrolysis of the TMS moieties with release of 5-azaC. The efficient release of the active from the prodrug was further confirmed when the GI_50_ of NUC041 and NUC013 in two cell lines were shown to be similar.

The pharmacokinetics of NUC041 formulated in LNE demonstrated a half-life of NUC013 unchanged from that of NUC013 administered IV. Additionally, the large Vss of NUC041 suggested that NUC041 might be sequestered, perhaps in the reticuloendothelial system or by concentrating in adipose tissue. When tested in a xenograft model of human colon cancer (LoVo), NUC041 demonstrated safety but only moderate efficacy; however, this was significantly less than reported for NUC013 in the same model in which significant tumor inhibition at equimolar doses was reported on study days 9 through 51 [9], as opposed to study days 27 through 30 for NUC041.

On this basis of these results, a decision was made to test a depot formulation that was designed to improve the half-life of NUC041. As opposed to a SQ route, the IM route of administration was selected to decrease the apparent volume of distribution in the event NUC041 was concentrating in adipose tissue, with subsequent release of NUC013. The IM route in the mouse is limited to a volume of 50 µL at one injection site. The half-life of NUC013 derived from NUC041 increased from 0.25 h in LNE to 3.4 h in PPD, an improvement of 13.6-fold; on a dose of equivalent basis, the AUCinf of NUC013 increased 4.1-fold.

Unfortunately, mice did not tolerate NUC041 formulated in PPD. Suspicion focused on complement activation related to the PEG moieties [17,18] or perhaps ethylene oxide [19,20], a known contaminant thereof, as NUC041 had been well tolerated when formulated in LNE and NUC013 has an MTD >120 mg/kg in mice [9]. That the vehicle was responsible for the observed toxicity was confirmed when weight loss was demonstrated with IM injection of vehicle alone. Additionally, that toxicity was compatible with a pseudoallergic reaction was confirmed when premedication with dexamethasone ablated all signs of toxicity. Dexamethasone in combination with other drugs has been successfully used for premedication prior to PEG-liposome infusions [21]. The dose of dexamethasone used in this study was selected on the basis of studies in mice of staphylococcal enterotoxin B [22]. The MTD for NUC041 formulated in PPD administered IM when mice were premedicated with dexamethasone was >3 mg/mouse or > a mean dose of 123.8 mg/kg. More frequent dosing than a weekly schedule of NUC041 with dexamethasone premedication was not tested because of the possibility of side effects from the steroid. In the absence of premedication, the MTD was established at 0.6 mg/mouse qod (mean of 25.7 mg/kg NUC041 qod at study initiation). 

The mouse xenograft model with human NCI-H460 subcutaneous implants has been shown to be robust and reproducible [23]. In this model, NUC013 administered for three consecutive days a week from study days 4 through 27 demonstrated a significant improvement in animal survival. The MS went from 25.5 days in animals administered SC to 38 days in mice administered NUC013. Likewise, tumor volume was significantly inhibited compared to SC on study days 14 through 27. Treatment with NUC041 formulated in PPD also showed significant tumor inhibition; however, this did not translate into a significant change in survival because of the development of tumor ulceration leading to per protocol euthanasia. It should be noted that increased tumor volume has been reported in response to immunotherapy in mice [24], as well as in humans, where it is referred to as pseudoprogression [25], which compromises the use of tumor volume as a measure of efficacy in the face of a robust inflammatory response. 

Mice treated with NUC041 developed significantly more ulceration (15 of 18 evaluable mice) than SC mice (4 of 10) (*p* = 0.035, Fisher’s exact test, two-tailed) or NUC013-treated mice (1 of 10) (*p* = 0.0003, Fisher’s exact test, two-tailed). Ulceration developed at significantly lower mean tumor volumes in both NUC041 groups: in the 3 mg NUC041 with dexamethasone group, mean tumor volumes were 1319 vs. 2770 mm^3^ for SC (*p* = 0.0043, Student *t*-test, two-tailed); while in the 0.6 mg NUC041 group, mean tumor volumes were 1474 vs. 2770 mm3 (*p* = 0.0068, Student *t*-test, two tailed). Finally, in the LoVo study, 2/10 mice had ulcerations when NUC041 was administered IV in LNE compared to 3/10 in SC mice. Hence, it is likely that the differentiating factor leading to tumor ulceration with NUC041 formulated in PPD was related to the prolonged exposure to NUC013, resulting in enhanced epigenetics effects. Such an outcome was not shown with NUC013 or NUC041 in LNE, where ulceration may simply have resulted from proliferative activity and hypoxia during tumor expansion [26]. This argument is further supported by the observation that tumors in mice treated with NUC013 or NUC041 in LNE grew more slowly than those in their respective SC mice and had even less ulceration than tumors in SC mice. There were four deaths over the four groups of animals. One each in the SC and NUC013 groups and two in the NUC041 0.6 mg group. The latter two deaths are noteworthy because the mice were found cannibalized, which may be linked to tumor ulceration.

The amount of NUC013 delivered to the three different groups in the NCI-H460 model on a weekly basis was (1) NUC013 20 mg/kg IV qd × 3, or 60 mg/kg/wk; (2) NUC041 2.4–3.0 mg IM qwk or a mean of 64.3–80.3 mg/kg/wk of NUC013; and (3) NUC041 0.6 mg IM qod or a mean of 58.2 mg/kg/wk of NUC013. This analysis assumes that all NUC041 was converted to NUC013. Reviewing the totality of the data generated in studies with NUC013 and NUC041, it is likely that the important metric for NUC013 efficacy is the length of time a tumor is exposed to NUC013 above a tumor-dependent threshold value.

5-aza nucleosides are thought to be S phase specific agents; however, it has been shown that cell cycle dependence is not absolute [27]. Nonetheless, cancerous cells in G1 and G2 may escape treatment with drugs with short half-lives, necessitating prolonged infusion. In the case of decitabine, the approved regimens in the United States require IV infusion of 15 mg/m^2^ over 3 h, repeated every 8 h for three days repeated every six weeks; or alternatively, infusion of 20 mg/m^2^ over 1 h for five days repeated every four weeks [5]. In a study of continuous infusion of over seven days in patients with refractory solid tumors, the MTD of decitabine was 2 mg/m^2^ qd, with neutropenia as the main dose limiting toxicity [7]. In a study of epigenetic priming of patients with acute myeloid leukemia, decitabine was administered by continuous infusion at a dose of 20 mg/m^2^ qd for up to seven days. Additional gastrointestinal toxicity was noted with seven days of treatment in comparison to treatments of five days or less [8]. Another approach to improve the pharmacokinetics of decitabine has been the development of a prodrug. Guadecitabine is a dinucleotide which consists of decitabine linked by a phosphodiester bond to deoxyguanosine. This linkage results in reduced susceptibility to immediate inactivation by CDA. However, following the cleavage of the phosphodiester bond, free decitabine can undergo deamination [28]. Guadecitabine has similar stability in an aqueous solution as decitabine and cytotoxicity is comparable [29]. In a Phase 1 clinical trial, the half-life guadecitabine was 0.59 to 1.44 h following subcutaneous administration and 1.23 to 1.79 h for decitabine derived from guadecitabine with efficient release of decitabine [30]. This was longer than the 35 min half-life of decitabine following IV infusion [5].

Contrary to other nucleoside DNMT inhibitors, such as decitabine, 5-fluoro-2′-deoxycytidine, or zebularine [31], NUC013 has been shown to be more effective against cancer cells expressing p53 wild type (WT) than p53 deficient cells [9]; both LoVo and NCI-H460 are p53 WT. p53 is often inactivated in cancer because it can trigger cell growth arrest, apoptosis, utophagy, or senescence which are detrimental to cancer cells, and it impedes cell migration, metabolism, or angiogenesis which are favorable to cancer cell progression and metastasis [32]. There are several genes that are known to be affected by hypermethylation in the NCI-H460 cell line in addition to p53. These include p73, a candidate tumor suppressor gene [33], and deleted in bladder cancer 1 (DBC1) gene whose restoration in NCI-H460 inhibits tumor growth [34]. Selected cancer testis antigens (CTA), Human Leukocyte Antigens (HLA), and accessory or co-stimulatory molecules required for efficient recognition of neoplastic cells by the immune system have been shown to be epigenetically silenced or down-regulated in cancer. DNMT inhibition induces expression of CTA and HLA class I antigens, which lasts several weeks after treatment [35]. For example, decitabine has been shown to cause sufficient expression of CTA in patients with acute myeloid leukemia for T-cell recognition [36]. CTA are known to be present in NCI-H460 [37]. 

The immune system of nude mice is characterized by a small population of T-cells, an antibody response confined to the IgM class, a low T cell-dependent response to antigens and an increased natural killer (NK) cell response. The population of mature CD8+ T-cell is cytolytically active [38]. Nude mice are good hosts for rapidly growing human solid tumor cell lines, but more immunosuppressed mice, e.g., SCID mice, are required for slower growing cell lines [39]. Because of the limited repertoire of acquired immunity, it is likely that the nude mouse model underestimates the effectiveness NUC013 would have in fully immunocompetent hosts following tumor epitope derepression and T-cell activation [40]. However, this does not preclude the possibility of a robust antitumor immune response induced by NUC013, leading to necrosis and tumor regression. Recent literature supports a relationship between p53 expression and NK cell activity. Indeed, p53 has been shown to be involved in NK cell functional maturation [41], and p53 induction in a NSCLC cell line resulted in upregulation of specific NKG2D ligands which enhanced NK cell-mediated target recognition [42]. As p53 or other tumor suppressor genes promote cellular senescence, these cells stop proliferating and mobilize an oncosuppressive mechanism mediated by immune cells. The senescence-associated secretory phenotype is associated with the release of chemokines such as CCL2 and cytokines, e.g., IL-2, IL-15 and IL-18, which are involved in the recruitment and activation of NK cells. Once activated, NK cells infiltrate neoplastic lesions and kill senescent cancer cells upon the recognition of NKG2D ligands displayed on their surface [43].

To the authors’ knowledge, this collection of findings has not previously been reported with DNMT inhibitor treatment in a tumor xenograft model, probably because other nucleosides are not very effective against p53 WT tumors and because drug exposure is too short to result in the induction of all the desired epigenetic effects. NUC041 formulated in PPD alters the balance between actively replicating malignant cells and cells undergoing apoptosis or senescence, ultimately resulting in tumor necrosis and regression. The predominantly neutrophilic inflammatory response was observed after approximately one month of treatment and is compatible with a response to cell death. When cells die, the tissue site is rapidly infiltrated with leukocytes, consisting initially of neutrophils, followed by the accumulation of monocytes [44]. In melanoma, ulceration has been found to be a favorable prognostic factor for response to a therapeutic vaccine [45] or PEG-interferon-α-2B [46]. The relevance of this clinical finding to the present study is unclear but warrants mentioning. 

NUC013 has demonstrated safety and efficacy in a mouse xenograft of NSCLC. NUC041 formulated in PPD resulted in prolonged exposure of tumor cells to NUC013, leading to a enhanced efficacy. The two mice in the NUC041 0.6 mg group (Mice 3 and 4) that were euthanized at 34 days, following the onset of tumor ulceration at 28 days, showed evidence of tumor regression as measured by a decrease in tumor volume of 38% to 51%. Furthermore, as evidenced by the photomicrographs, these tumors included a substantial area of ulceration and necrosis, leaving only a small area of neoplasia. However, vehicle toxicity or possible side effects of steroid pretreatment precluded higher doses or more frequent administration of NUC041, possibly preventing even better efficacy in this model. Also, had treatment continued of the ulcerated tumors, it is possible that the outcome would have been cures. 

The half-life of drugs in depot formulations is partially dependent on the volume of infusate [47]. In mice, the limit for IM injections is a volume of 50 µL at a single site. However, substantially larger volumes could be administered to humans IM or SQ; consequently, it is likely that the half-life of the drug would be significantly more prolonged, perhaps by days, were a better tolerated derivative of the current formulation developed. It is also noteworthy that NUC041 formulated in PPD has been shown to be stable for >2 months at 4 °C (Supplementary Table S1). Other approaches also could be taken, such as packaging NUC041 in a pegylated liposome. Hydrophobic drugs can be typically be packaged in the lipid bilayer [48] and drug half-life of the order of 50 h in humans has been achieved [49]. Further formulation development will be necessary to optimize the various pharmacokinetic parameters and minimize vehicle-related toxicity. 

## 4. Materials and Methods

### 4.1. NUC041 Synthesis

NUC013, NUC025 and NUC041 were originally synthesized at Sonus Pharmaceuticals (Bothell, WA, USA); subsequently, NUC013 and NUC041 were synthesized by NuChem Therapeutics (Montreal, QC, Canada).

NUC041 can be prepared from 2′,2′-difluoro-5-azadeoxycytidine by treating 2′,2′-difluoro-5-azadeoxycytidine with excess of 1,1,1,3,3,3-hexamethyldisilazane and catalytic amounts of ammonium sulfate at 125 °C. NUC041 can then isolated by flash chromatography. Compound characterization was provided by NMR in Supplementary Figure S1. The compound was >97% pure. 

### 4.2. In Vitro Activity

As per methods previously presented [9], cells were grown in an appropriate medium for the cell line of interest. 96 well plates of each cell line were seeded with 5000 cells per well and left overnight. Drug-exposed cells were incubated at 37 °C for 72 h. At the end of the 72-h exposure period, plates were removed for the CellTiter-Glo^®^ assay (Promega, Madison, WI, USA). Luminescence was recorded on a Synergy 4.0 (BioTek Instruments, Winooski, VT, USA). Assays were performed in triplicate.

### 4.3. Pharmacokinetic Studies

These studies were carried out in strict accordance with the recommendations of the NIH Guide for the Care and Use of Laboratory Animals. The protocols were approved by the IACUC of Southern Research Institute (Birmingham, AL, USA) (AAALAC Accreditation: 000643, IACUC approval number 15-03-009B).

Quantitative determination of NUC013 and NUC041 in quenched mouse whole blood was accomplished using protein precipitation and high-performance liquid chromatography with tandem mass spectrometry detection. Gemcitabine-^13^C, ^15^N_2_ “GemC-^13^C”, was used as the internal standard. NUC013, NUC041 and GemC-^13^C were extracted from 50 µL whole blood using protein precipitation with acetonitrile. Extracts were analyzed by hydrophilic interaction chromatography at 35 °C, using a XBridge™ Amide column (Waters Corp., Milford, MA, USA) under isocratic conditions, with 100 mM ammonium acetate pH 9: ethanol: acetonitrile (25:25:500, *v*/*v*/*v*) as mobile phase A. Column effluents at a constant flow rate of 300 µL/min were analyzed by multiple reaction monitoring (MRM) using a triple quadrupole mass spectrometer in positive-ion mode (AB Sciex 4000 Q Trap, equipped with TurboV IonSpray^®^) (SCIEX, Framingham, MA, USA). The precursor/product transitions were 265^®^113 *m*/*z* for NUC013, 409^®^185 for NUC041 and 267^®^115 *m*/*z* for GemC-^13^C.

The calibration curve for each analyte was fitted using weighted (1/x^2^) linear regression analysis of the Analyte/IS peak area ratio versus Analyte/IS concentration ratio from 5–5000 ng/mL. Concentrations of incurred and quality control samples were calculated with the same regression analysis and results were reported in ng/mL of analyte. 

For the study of NUC041 formulated in LNE, blood was collected at 3, 6, 15, 30, 45, 60, 120, and 240 min. For the study of NUC041 formulated in PPD, blood was collected at 0.25, 0.5, 1, 2, 4, 8, 12, and 18 h. Pharmacokinetic parameters were calculated from mean concentrations of NUC-041 or NUC013 in blood over time for mice using Phoenix^®^ WinNonlin^®^ (Version 6.3; Pharsight, A Certara Company; Cary, NC, USA).

### 4.4. Tumor Xenograft Studies

As per methods previously presented [9], the tolerability studies and the tumor xenograft studies were performed at the same institution. These studies were carried out in strict accordance with the recommendations of the NIH Guide for the Care and Use of Laboratory Animals. The protocols were approved by the IACUC of Southern Research Institute (Birmingham, AL, USA) (AAALAC Accreditation: 000643, IACUC approval number 15-03-009B).

10^7^ tumor cells from culture in Matrigel™ of human LoVo colon cancer cells were subcutaneously implanted in the flank of 1.75-fold the number of NCr-nu/nu mice required for the study. Study initiation began when the required number of mice had tumors of approximately 40 to 75 mm^3^. Similarly, 10^7^ tumor cells from culture in Matrigel™ of NCI-H460 human NSCLC were subcutaneously implanted in the flank of 1.75-fold the number of NCr-nu/nu mice required for the study. Study initiation began when the required number of mice had tumors of approximately 32 to 75 mm^3^. 

Mice with tumors in the proper volume range were arbitrarily assigned to groups. Mice received test article as described in the text. Mice were observed daily for mortality and moribundity with weights and the tumor measurements taken twice weekly. Tumor volume was determined using the formula for an ellipsoid sphere: Length × Width^2^/2 = Volume (mm^3^). The experiments were scheduled to last 60 days from the day of tumor implant. Any animal whose weight decreased more than 30% from the weight on the first day of treatment or whose tumor reached 4000 mm^3^ in volume, ulcerated, sloughed off, or was moribund was euthanized prior to study termination. Per protocol amendment, two mice in the NUC041 0.6 mg group continued to receive treatment for four days following ulceration before euthanasia.

### 4.5. Formulations

Each contained an oil phase consisting of an injectable oil and lecithin and an aqueous phase.

As per methods previously presented [23], the lipid nano-emulsion (Latitude Pharmaceuticals, San Diego, CA, USA) contained an oil phase consisting of an injectable oil and lecithin and an aqueous phase comprising a tonicity adjuster, a stabilizer, and water. The emulsions were of oil-in-water type with the mean diameter of oil droplets <100 nm. The vehicle was at neutral pH (5–7) and about isotonic and stored at 4–8 °C. 

The PEG-phospholipid depot (Latitude Pharmaceuticals, San Diego, CA, USA) comprised of S-100, DSPE-PEG 2000, sesame oil, and alcohol. These four constituents were placed in a vial, warmed to 40 °C until all products dissolved, and the vehicle was then vortexed. Subsequently, the vehicle was filtered through a 0.22 µm syringe filter and NUC041 was added. The formulated product was heated to 40 °C, vortexed, and stored at 2–8 °C. 

### 4.6. Histology

Approximately half of each tumor was fixed in 10% neutral-buffered formalin and the other half was frozen. The fixed neoplasm was trimmed, processed, embedded, and microtomed (approximately 5 µm sections). Tissue sections were mounted on glass slides, stained with hematoxylin and eosin (H&E), and coverslipped. Slides were submitted to a veterinary pathologist for a histopathologic evaluation. 

## 5. Conclusions

Previously, NUC013 had been shown to be effective in a model of human colon cancer [9]. In this study, the activity of NUC013 has been extended to successfully treat another solid tumor, human NSCLC. However, NUC013, as all approved DNMT inhibitors or those under clinical development, suffers from a short half-life which is problematic in a drug targeting primarily tumor cells in S phase. NUC041 has demonstrated that, when properly formulated, a dramatically increased half-life of the active, NUC013, is possible. In turn, that prolonged half-life resulted in a different outcome for mice treated with NUC013 derived from NUC041 when compared to IV NUC013. Tumors in mice treated with NUC041 have shown tumor ulceration, necrosis, and tumor regression that is compatible with p53 derepression and activation of the immune system. Further formulation development will be necessary to optimize the pharmacology of NUC013 derived from NUC041 and minimize vehicle toxicity. 

## Figures and Tables

**Table 1:** Comparison of GI_50_ between NUC013 and its prodrug, NUC041.

Compound	NSCLC NCI-H460 GI_50_ (µM)	Colon Cancer HCT-116 GI_50_ (µM)
NUC013	1.57	2.58
NUC041	1.61	2.80

**Table 2:** Pharmacokinetic parameters calculated from concentrations of NUC041 and NUC013 in whole blood following IV administration of NUC041 (15 mg/kg) to mice in LNE vehicle. Where: C_max_: Maximum observed concentration in blood. T_max_: (1) NUC013: Time of maximum observed concentration in blood; (2) NUC041: First sampling time. T_1/2_: Half-life of the terminal elimination phase. AUC_last_: Area under the blood concentration versus time curve from time 0 to the last sampling time the analyte was quantifiable in blood. AUC_inf_: Area under the blood concentration versus time curve from time 0 to infinity. Cl: Total body clearance. Vss: Volume of distribution at a steady state.

Analyte	C_max_ (ng/mL)	T_max_ (min)	T_1/2_ (min)	AUC_last_ (h·ng/mL)	AUC_inf_ (h·ng/mL)	Cl (mL/min/kg)	Vss (mL/kg)
NUC041	2803	3	11	580.4	590.6	423	4431
NUC013	745	15	15	464.0	468.0	NA	NA

**Table 3:** Pharmacokinetic parameters derived from mean concentrations of NUC-041 and NUC-013 in whole blood following IM administration of NUC-041 (3 mg/mouse) formulated in PPD. Abbreviations as per Table 2, except MRT: Mean residence time. Vz/F: Apparent volume of distribution during terminal phase after non-intravenous administration.

Analyte	C_max_ (ng/mL)	T_max_ (h)	T_1/2_ (h)	AUC_last_ (h·ng/mL)	AUC_inf_ (h·ng/mL)	MRT (h)	Vz/F (mL/kg)
NUC041	4210	0.5	1.7	6030	6261	2.6	1172
NUC013	1333	1	3.4	5629	5813	5.1	NA

**Table 4:** Characteristics of tumors in mice treated with NUC041 and histologic findings.

Mouse Identifier	NUC041 Dose & Regimen	Study Day of Scheduled Euthanasia	Tumor Volume (mm^3^)/Ulceration	Histopathology
1	2.4/3.0 mg/mouse qwk + dexamethasone	35	2746/no ulceration	30–40% necrosis
2	0.6 mg/mouse qod	34	3035/no ulceration	50–60% necrosis
3	0.6 mg/mouse qod	34	1960/ulceration	10% necrosis *
4	0.6 mg /mouse qod	34	936/ulceration	5% necrosis *

* Assessment of percent necrosis excludes area of ulceration.

## Supplementary Materials

The following are available online at http://www.mdpi.com/1424-8247/11/2/36/s1, Table S1: Study of the stability of NUC041 formulated in PPD stored at 2–8 °C for 60 days, as measured by HPLC. Figure S1: ^1^H NMR of NUC041.
