# Supplementary material for: NUC041, a Prodrug of the DNA Methytransferase Inhibitor 5-aza-2′,2′-Difluorodeoxycytidine (NUC013), Leads to Tumor Regression in a Model of Non-Small Cell Lung Cancer
Source: Pharmaceuticals (Basel). 2018 Apr 23;11(2):36. doi: 10.3390/ph11020036 (PMC6027359; doi:10.3390/ph11020036)
Supplement: Supplementary file 1 [file pharmaceuticals-11-00036-s001.pdf]

Supplementary Materials

Table S1: Study of the stability of NUC041 formulated in PPD stored at 2-8°C for 60 days, as measured by HPLC.

ID	Assay (mg/mL)	% Recovery vs assay time 0
NUC041 in PPD time 0	16.24	-
NUC041 in PPD day 60	16.05	98.8

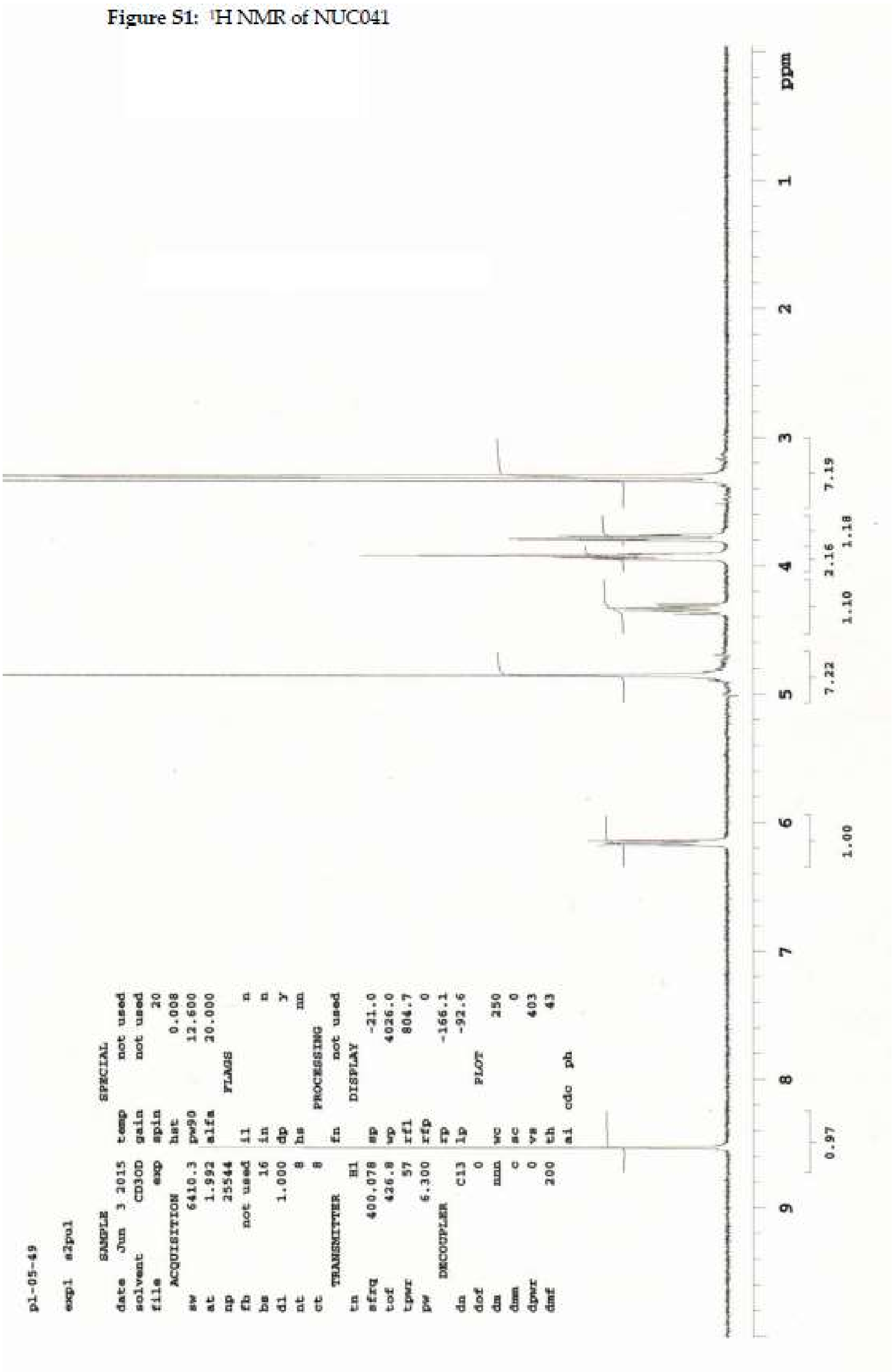

Figure S1: NUC041 formulated in PPD is stable when it is stored at 2-8°C for 60 days. The recovery at this storage condition for 60 days is approximately 98.8%.
